# Unrecognized extensive charge of microbial gas in the Junggar basin

**DOI:** 10.1038/s41598-024-62706-8

**Published:** 2024-05-25

**Authors:** Hao Zhang, Chunfang Cai, Xiaomin Mei, Daowei Wang, Dawei Liu, Erting Li, Jun Jin, Menglin Zheng, Yong Tang

**Affiliations:** 1 Key Laboratory of Cenozoic Geology and Environment, Institute of Geology and Geophysics, Chinese Academy of Sciences, Beijing, 100029 People’s Republic of China; 2 Innovation Academy for Earth Science, Chinese Academy of Sciences, Beijing, 100029 People’s Republic of China; 3 College of Earth and Planetary Sciences, University of Chinese Academy of Sciences, Beijing, 100049 People’s Republic of China; 4 Yulin University, Shaanxi, 719000 People’s Republic of China; 5 Petroleum Exploration and Production Research Institute of SINOPEC, Beijing, 100083 People’s Republic of China; 6 PetroChina Xinjiang Oilfield Company, Xinjiang, 834000 People’s Republic of China

**Keywords:** Microbial gas, Methanogenesis, Mixing, Gas accumulation, Junggar basin, Mahu sag, Natural gas, Energy

## Abstract

Different from the Qaidam basin with about 320 billion m^3^ microbial gas, only limited microbial gases were found from the Junggar basin with similarly abundant type III kerogen. To determine whether microbial gases have not yet identified, natural gas samples from the Carboniferous to Cretaceous in the Junggar basin have been analyzed for chemical and stable isotope compositions. The results reveal some of the gases from the Mahu sag, Zhongguai, Luliang and Wu-Xia areas in the basin may have mixed with microbial gas leading to straight ethane to butane trends with a “dogleg” light methane in the Chung’s plot. Primary microbial gas from degradation of immature sedimentary organic matter is found to occur in the Mahu sag and secondary microbial gas from biodegradation of oils and propane occurred in the Zhongguai, Luliang and Beisantai areas where the associated oils were biodegraded to produce calcites with δ^13^C values from + 22.10‰ to + 22.16‰ or propane was biodegraded leading to its ^13^C enrichment. Microbial CH_4_ in the Mahu sag is most likely to have migrated up from the Lower Wuerhe Formation coal-bearing strata by the end of the Triassic, and secondary microbial gas in Zhongguai and Beisantan uplifts may have generated after the reservoirs were uplifted during the period of the Middle and Late Jurassic. This study suggests widespread distribution of microbial gas and shows the potential to find large microbial gas accumulation in the basin.

## Introduction

Microbial gas is characterized by a high dryness coefficient with C_1_/C_1–5_ > 0.95 and is enriched in ^12^C with δ^13^C_1_ <  − 55‰, and it accounts for 8 ~ 15% of the world's gas^1,2^. Microbial gas includes primary and secondary ones. Primary microbial gas is a product of organic matter in immature stage (*R*o ≤ 0.5%) by biochemical degradation of organic matter via acetate fermentation or CO_2_ reduction^3,4^. In general, microbial gas is generated through CO_2_ reduction in marine sediments, in which acetate is largely consumed by sulfate-reducing bacteria. Compared to acetate fermentation, CO_2_ reduction is the major pathway that forms commercial microbial gas accumulations^5,6^.

Secondary microbial gas is derived from degradation of oils and ethane and all higher n-alkanes^7^, and is generally related to tectonic uplift and has methane δ^13^C values < − 55‰ and the associated CO_2_ δ^13^C >  + 2.0‰. These values are shown to relate to oil biodegradation levels and proportions of reduced CO_2_^8^. Milkov^1^ systematically summarized the geological and geochemical characteristics of secondary microbial methane: (1) associated with biodegradable crude oil; (2) C_1_/∑C_1_–C_5_ is higher than 95%; (3) δ^13^C_1_ <  − 55.0‰ ~  − 35.0‰; (4) δ^13^C_CO2_ >  + 2.0‰; (5) reservoir temperature is < 70 °C and occasionally up to ~ 90 °C.

The chemical and stable isotope compositions of natural gas have been used to distinguish between microbial and thermogenic gases in the subsurface^2,5,9–11^. Thermogenic gases typically have methane ^13^C_1_ values between − 55‰ and − 25‰ although early mature thermogenic gas can have methane δ^13^C values as light as − 73‰, and relatively high concentrations of C_2_^+^ hydrocarbons^2,5^. Microbial gas is dominantly composed of methane with δ^13^C values lighter than − 55‰ or less^2,5^.

Natural gas has been found from different tectonic units around Junggar basin, but the commercial natural gas distributes mainly in the eastern Luliang area in the east of the basin, and Hutubi, Manas, and Horgos anticlines in the southern margin of the basin. Natural gas in the NW Junggar basin is considered to have been thermally cracked from both types I and III kerogen and their derived oils^12^. The natural gas in the Mahu Sag was derived from the source rocks of the Lower Permian Fengcheng Formation with present *R*o = 0.85 ~ 1.16%^13,14^. Microbial gas is reported to limit to Shazhang area in the eastern Junggar Basin^15^, based on relationships of δ^13^C_1_ and δ^13^C_2_, δ^13^C_1_ and C_1_/(C_2+3_). That is, microbial gas in the Junggar basin shows limited distribution, which is much less than a proven reserve of about 320 billion m^3^ in Qaidam basin with similarly abundant type III kerogen^16^. Interestingly, extremely negative δ^13^C values (− 70 to − 22‰) from high Mn calcites (average MnO = 5 wt%) have been reported from Lower Triassic Baikouquan Formation sandy conglomerates in the Mahu sag^17^, leading to a possibility for the oxidized methane to have been microbial^18,19^. In Zhongguai and Beisantai uplifts, some oils have been biodegraded, and some Fe- or Mn-rich calcite shows δ^13^C values of about + 22‰^20^, secondary microbial gases may have occurred. Thus, it is necessary to analyze gases from Mahu sag, Zhongguai and Beisantai uplifts, and other parts of the basin to determine if they are microbial and hopefully to find new microbial gas pools in these areas and provide some clues to identifying microbial gas in the basin.

## Geological setting

**Figure 1:**
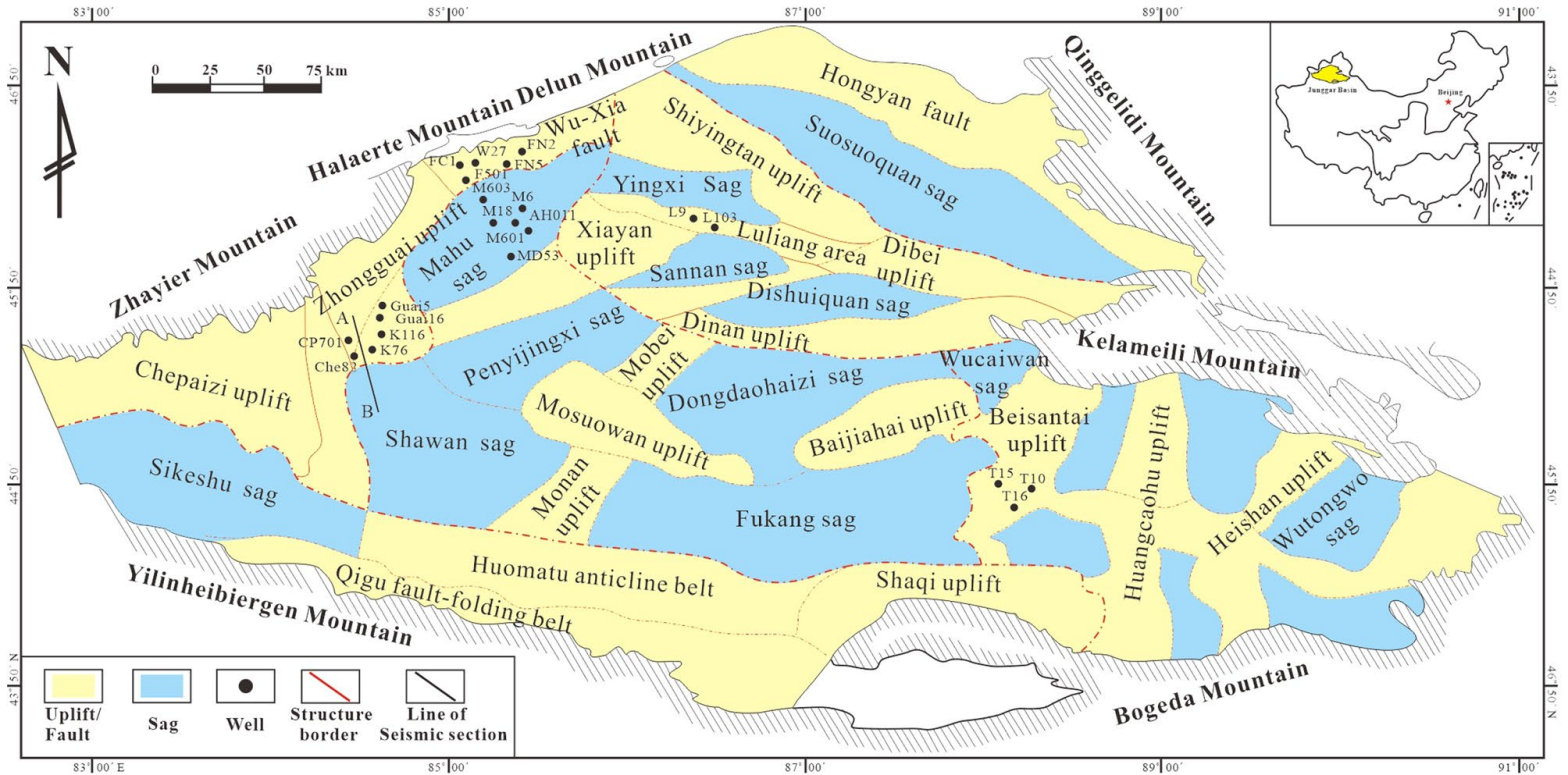
Diagram showing division of structural units in Junggar Basin and well locations for gas samples (created with CoreDRAW × 7, http://www.coreldraw.com/en/).

The Junggar Basin is one of the three major superimposed petroliferous basins in western China, which is rich in oil and gas resources, covering an area of ~ 1,34,000 km^2^, and is bounded to the northwest by the Zhayier and Halalate Mountains, to the northeast by the Kelameili and Qinggelidi Mountains, and to the south by the Yilinheibiergen and Bogeda Mountains of the Tianshan Range^21^ (Fig. 1). It is an upper Paleozoic, Mesozoic, and Cenozoic superimposed basin, located at the junction of the Kazakhstan, Siberia and Tarim blocks. The basin has experienced many stages of evolution, such as the extinction and collision orogeny of the paleo Asian Ocean from the Middle Ordovician to the Early Carboniferous, the extension and fault depression from the Late Carboniferous to the early Permian, transformation of the fault depression from the middle to the Late Permian, the unification of the depression during the Mesozoic and the formation of intracontinental foreland during the Cenozoic. According to its geotectonic position, variations in basement rocks, and structural evolution, the basin can be subdivided into six structural units^22^ (Fig. 1).

**Figure 2:**
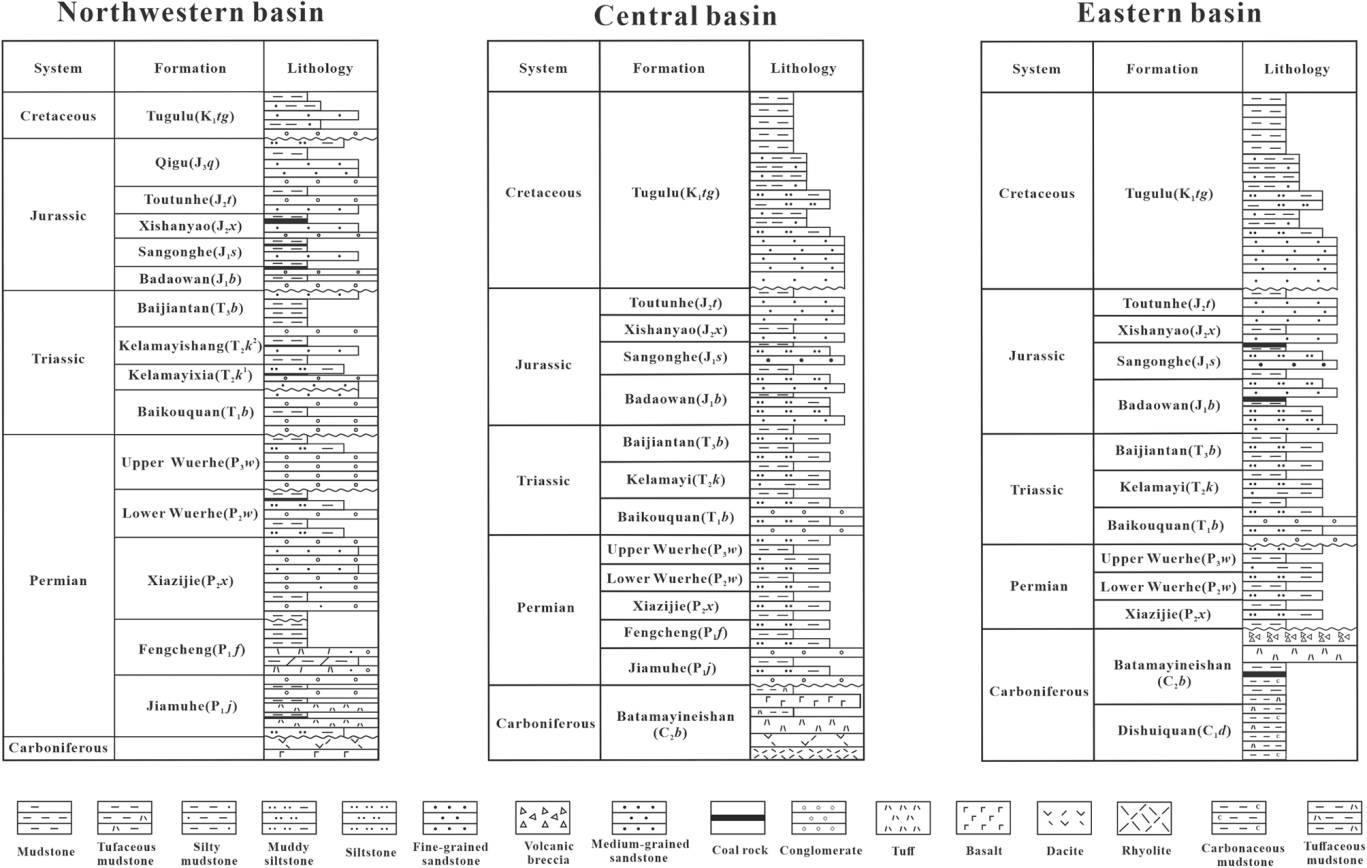
The stratigraphic sequence of the Junggar Basin. The stratigraphy is based on refs.^12,15,30^.

In the Junggar Basin, there are several sets of source rocks, including the Carboniferous, Lower Permian Jiamuhe Formation and Jurassic high-quality type III, gas-prone source rocks; and the Lower Permian Fengcheng Formation, Middle Permian Lower Wuerhe Formation high-quality oil-prone source rocks^23,24^(Fig. 2). Most of the hydrocarbon accumulation in the basin were derived from the above three suites of the source rocks in terms of the source rock distribution and hydrocarbon generation history^25^. Three major hydrocarbon-bearing systems were formed with Carboniferous and Permian oil and gas source rocks dominating in the eastern basin, Permian oil source rocks in the central and western parts, and Jurassic gas source rocks in the south. The Carboniferous source rocks are mainly tuff mudstones, with an average TOC content of 1.2%, an average hydrocarbon generation potential (PG = S_1_ + S_2_) of 0.75 mg/g. The source rock is high maturity (vitrinite reflectance *R*o > 0.9%) type III kerogen and thus was favorable for natural gas generation^26^. The industrial-scale source rocks are mainly located in the Dishuiquan sag on the Luliang area.

Lower Permian to Middle Permian Fengcheng Formation (P_1*f*_), Lower Wuerhe Formation (P_2_*w*), the Pingdiquan Formation (P_2_*p*) and the Lucaogou Formation (P_2_*l*) mudstones were deposited in a deep anoxic lacustrine environment and are the most important source rocks in the basin^27^. The P_1*f*_ source rocks are mostly mature to highly mature (*R*o = 0.85 ~ 1.16%) lacustrine mudstones with an average TOC of 1.38% and an average S_1_ + S_2_ of 5.6 mg /g, and a Type I ~ II kerogen^28^. Burial history rebuilding shows that the source rocks have experienced *R*o of ≤ 2% in the depression center, indicating that the source rocks are in the stage of oil to wet gas generation. This P_1*f*_ may have contributed billion-tonne oil reserves in the Mahu area^29^.

The Middle Permian source rocks show a significantly wider distribution than the P_1*f*_ and are widely distributed in the central depression of the basin and in the eastern uplift sedimentary depressions. In the northwest margin and central part of the basin, the P_2_*w* source rocks are mainly type III kerogen, have a TOC range from 1.4% to 1.73%, S_1_ + S_2_ of 2.68 mg/g, and *R*o values of 0.64% ~ 1.56%^23,24^, indicating that the organic matter is within the mature to high mature stage. The P_2_*l* source rocks are distributed along the south margin dominated by oil shale and black-grey mudstone, with an average TOC of 7.60%, and S_1_ + S_2_ of 34.95 mg/g. The organic matter is mainly low to mature Type II and Type I kerogen, with *R*o values ranging from 0.50% to 0.91%^25^.

Hydrocarbon production peaks and charge have been considered to occur during T_3_–J_2_, K_1_, K_2_–E and N–Q, which correspond to the tectonic development periods T_3_, J_3_, K and N–Q^28^.

## Materials and methods

24 gas samples produced from Carboniferous to Cretaceous were collected (Table 1), including 6 samples from the Mahu sag, 7 samples from the Wu-Xia fault, 6 samples from the Zhongguai uplift in the western Shawan sag, 2 samples from the Luliang area and 3 samples from the Beisantai uplift (Fig. 1). The Mahu sag, Wu-Xia fault and the Zhongguai uplift are in the northwest of the Junggar basin, the Luliang area is in the central and the Beisantai uplift is in the east of the basin. All gas samples were collected from the wellhead in the commercial petroleum production field, after flushing the bottles for 15–20 min to remove air contamination. Double-ended, high-grade stainless steel bottles equipped with shut-off valves were used to collect gas samples.

**Table 1:** Chemical composition and carbon and hydrogen isotope data of natural gas from the Junggar Basin.

Area	Well	Formation	Depth (m)	Gas chemical compositions (%)							Dryness coefficient	C_1_/(C_2_ + C_3_)
				C_1_	C_2_	C_3_	C_4_	C_5_	N_2_	CO_2_		
Zhongguai uplift	Guai5	P_2_*x*	3116–3126	93.61	2.10	0.71			2.61	0.27	0.97	33.31
	Guai16	J_1_*s*	1534–1838	94.59	0.23				5.03		1.00	411.26
	K76	P_2_*w*	2964.6	91.52	4.78	1.76			0.82		0.93	13.99
	K116	C	524–550	94.04	2.34	1.50	0.77	0.10	1.15	0.03	0.95	24.49
	Che82	J_1_*b*		95.79	1.79	0.30	1.08		0.24		0.97	45.83
	CP701	J_1_*b*		92.32	1.70	0.27	0.46		2.00		0.98	46.86
Wu-Xia fault	FC1	P_1*f*_	3855	69.89	5.52	2.56	1.45	1.65	11.50	4.04	0.86	8.65
	W27	P_2_*x*	2311	89.81	3.13	2.11			0.23	1.92	0.94	17.14
	F501	P_1*f*_	972	78.36	6.65	5.88	3.54	1.75	2.50		0.81	6.25
	FN2-1	P_1*f*_	4037.8	83.96	6.96	2.96			4.49		0.89	8.46
	FN5-1	P_1*f*_	4394.2	75.24	11.27	6.91	3.44	1.46	0.61	0.16	0.77	4.14
	FN2-2	P_1*f*_	4037.8	83.25	5.39	2.22			7.66		0.92	10.94
	FN5-2	P_1*f*_	4418	64.68	10.2	7.21	4.38	3.28	2.02	0.35	0.72	3.72
Mahu sag	M18	T_1_*b*	3854	86.08	5.23	2.29	2.14	0.76	2.19	0.78	0.89	11.45
	M6	T_1_*b*	3880	84.38	6.23	3.10	3.39	1.56	0.70	0.09	0.86	9.04
	AH011	T_1_*b*	3848	85.93	5.06	2.18	2.06	0.89	3.14	0.22	0.89	11.87
	M601	T_1_*b*	3893	88.53	4.27	1.29	0.89	0.32	4.42	0.10	0.93	15.92
	M603	T_1_*b*	3883	88.10	0.98	0.34	0.26	0.11	9.09	1.06	0.98	66.74
	MD53	T_1_*b*	3860	86.14	4.99	2.02	1.92	0.85	3.16	0.50	0.90	12.29
Luliang area	L9	K_1_*tg*	1186–1192	93.54	3.34	0.44			1.96	0.00	0.96	24.75
	L103	K_1_*tg*	1227.5–1230	94.99	0.14				4.79	0.08	1.00	678.50
Beisantai uplift	T15	J_3_*q*	2011–2032.5	97.03							0.98	
	T10	J_3_*q*	2023–2046	98.34							0.99	
	T16	J_3_*q*	1920.5–1974	96.54							0.98	

Area	Well	Formation	δ^13^C (‰, VPDB)					δ^2^H (‰, VSMOW)				
			C_1_	C_2_	C_3_	C_4_	CO_2_	δ^2^H _C1_	δ^2^H _C2_	δ^2^H _C3_	δ^2^H _*i*C4_	δ^2^H _*n*C4_
Zhongguai uplift	Guai5	P_2_*x*	− 39.6	− 26.8	− 24.4	− 26.8						
	Guai16	J_1_*s*	− 56.0	− 25.6								
	K76	P_2_*w*	− 40.5	− 29.8	− 30.5	− 29.3						
	K116	C	− 49.1	− 31.9	− 30.7	− 29.7						
	Che82	J_1_*b*	− 44.1	− 26.6								
	CP701	J_1_*b*	− 42.3	− 27.8	− 25.1							
Wu-Xia fault	FC1	P_1*f*_	− 52.7	− 37.5	− 34.0	− 33.6						
	W27	P_2_*x*	− 48.9	− 35.4	− 32.9	− 31.9						
	F501	P_1*f*_	− 53.0	− 40.9	− 37.7	− 34.6						
	FN2-1	P_1*f*_	− 52.5	− 39.6	− 35.0	− 33.2						
	FN5-1	P_1*f*_	− 54.4	− 38.6	− 35.1	− 33.5						
	FN2-2	P_1*f*_	− 41.4	− 25.1	− 22.0	− 24.3						
	FN5-2	P_1*f*_	− 54.2	− 37.9	− 34.5	− 36.8						
Mahu sag	M18	T_1_*b*	− 42.8	− 30.8	− 28.6	− 27.7						
	M6	T_1_*b*	− 46.8	− 31.9	− 28.7	− 28.7						
	AH011	T_1_*b*	− 43.1	− 31.0	− 29.2	− 28.3						
	M601	T_1_*b*	− 43.4	− 31.0	− 28.8		− 23.7	− 169.7	− 188.2	− 181.6	− 214.8	− 181.0
	M603	T_1_*b*	− 41.1	− 30.7	− 31.3		− 20.3	− 151.2	− 182.4	− 170.8		
	MD53	T_1_*b*	− 42.4	− 32.5	− 31.9		− 20.9	− 160.3	− 189.0	− 170.9	− 204.3	− 184.6
Luliang area	L9	K_1_*tg*	− 50.1	− 28.0	− 14.8	− 19.0						
	L103	K_1_*tg*	− 54.8	− 24.5								
Beisantai uplift	T15	J_3_*q*	− 50.7	− 28.4								
	T10	J_3_*q*	− 51.4	− 31.4								
	T16	J_3_*q*	− 49.8	− 28.7								

The chemical composition of the gas samples has been analyzed by a combination of mass spectrometry and gas chromatography. The analysis of C_1_ to C_5_ hydrocarbons was carried out using an Agilent 6890 N gas chromatograph equipped with a flame ionization detector. The individual gas components of hydrocarbons were separated through a capillary column (PLOT Al_2_O_3_ 50 m × 0.53 mm). The GC oven temperature was initially set at 30 °C for 10 min, and then gradually increased by 10 °C/min until it reached 180 °C. After reaching this temperature, it was maintained at this level for 20–30 min. Using a Finnigan MAT-271 mass spectrometer, non-hydrocarbon gases were determined. Using the calibration curve obtained from standard gases, the concentration of each component was determined. The data of the non-hydrocarbon components obtained from the mass spectrometer and the data of the hydrocarbon gas component (C_1_ to C_5_) obtained from the gas chromatograph were normalized to obtain the final results of the complete component data.

The stable carbon isotope ratios were measured using a Trace 1310 gas chromatograph coupled with a Thermo Finnigan Delta V Advantage mass spectrometer. The GC conditions for the carbon isotope were as follows: A HP-PLOT-Q column measuring 30m × 0.53mm × 40μm was used. The carrier gas, Helium (99.999%), flowed at a rate of 3.0 ml/min. The GC oven temperature was increased from 50 °C to 200 °C at a rate of 15 °C/min and maintained at 200 °C for 20 min. We used a split injection mode with a split ratio of 6 and an injector temperature of 200 °C. The temperature of the oxidation furnace was set at 975 °C. Carbon stable isotope ratios are expressed in δ notation in permil (‰) relative to the Vienna Pee Dee Belemnite (V-PDB) standard.

Hydrogen isotopic compositions were measured using a Thermo Delta V Advantage instrument interfaced with a Trace 1310 gas chromatograph. The GC was equipped with a 50 m × 0.53 mm HP-Al_2_O_3_/KCl column coated with a 10 μm film. The helium carrier gas flowed at a rate of 3.0 ml/min. The GC oven was held constant at 45 °C for 3 min and then heated to 200 °C at a rate of 15 °C/min and held at 200 °C for 20 min. The sample was injected using split mode with a split ratio of 6 and an injector temperature of 200 °C. The cracking furnace temperature was set to 1460 °C, and the H_3_^+^ factor was tested at least once a day with a value of less than 10 ppm/nA. The analyses are reported relative to the standard mean ocean water (V-SMOW) standard.

## Results

### Chemical composition of the gas

All the gases analyzed are dominated by hydrocarbon gas with contents from 81.07% to 98.96% and non-hydrocarbon gas including N_2_ and CO_2_ with contents from 0.23% to 11.50% and 0.08 to 4.04%, respectively (Table 1). The gases from the Mahu sag have dryness coefficients (C_1_/∑C_1_ − C_5_) from 0.86 to 0.98, N_2_ and CO_2_ contents from 0.70% to 9.09% and 0.09 to 1.06%, respectively. The gases from the Wu-Xia fault have dryness coefficients from 0.72 to 0.94, N_2_ and CO_2_ contents from 0.23% to 11.50% and 0.16 to 4.04%, respectively. The gases from the Zhongguai uplift have dryness coefficients from 0.93 to 1, N_2_ and CO_2_ contents from 0.24% to 5.03% and 0 to 0.27%, respectively. The dryness of gas in the Luliang area and Beisantai uplift is higher, from 0.96 to 1 and 0.98 to 0.99 respectively. Among different regions of the study area, the dryness coefficients decrease from the Beisantai uplift (average 0.98) to the Luliang area (average 0.98), the Zhongguai uplift (average 0.97), the Mahu sag (average 0.91), and the Wu-Xia fault (average 0.84).

### Isotopic composition of the gases

The gas samples of the Junggar basin show a wide variation of the stable carbon and hydrogen isotopic compositions. The δ^13^C values of methane, ethane, propane, and butane in the Mahu sag range between − 46.8‰ and − 41.1‰, between − 32.5‰ and − 30.7‰, between − 31.9‰ and − 28.6‰, and between − 28.7‰ and − 27.7‰, respectively. The carbon dioxide from the Mahu sag shows δ^13^C values ranging from − 20.3‰ to − 23.7‰ (Table 1), with an average of − 21.6‰. The gases have methane δ^2^H values from − 169.7‰ to − 151.2‰, with an average of − 160.4‰. The δ^13^C values of methane, ethane, propane, and butane in the Zhongguai uplift range between − 56.0‰ and − 39.6‰, between − 31.9‰ and − 25.8‰, between − 30.7‰ and − 24.4‰, and between − 29.7‰ and − 26.8‰, respectively. The δ^13^C values of methane, ethane, propane, and butane in the Wu-Xia fault range between − 54.4‰ and − 41.4‰, between − 40.9‰ and − 25.1‰, between − 37.7‰ and − 22.0‰, and between − 36.8‰ and − 24.3‰, respectively. As for the Luliang area, the δ^13^C values of methane, ethane, propane, and butane range between − 54.8‰ and − 50.1‰, between − 28.0‰ and − 24.5‰ respectively. Gas samples from the Beisantan uplift have δ^13^C values of methane, and ethane range between − 50.7‰ and − 49.8‰, between − 31.4‰ and − 28.4‰ respectively. The δ^13^C value of methane in the Junggar Basin is the lightest in the Luliang area (average − 52.5‰), followed by the Wu-Xia fault (average − 51.0‰), Beisantai uplift (average − 50.6‰), Zhongguai uplift (average − 45.3‰), and heaviest in the Mahu sag (average − 43.3‰).

## Discussion

### Evidence of microbial gas

**Figure 3:**
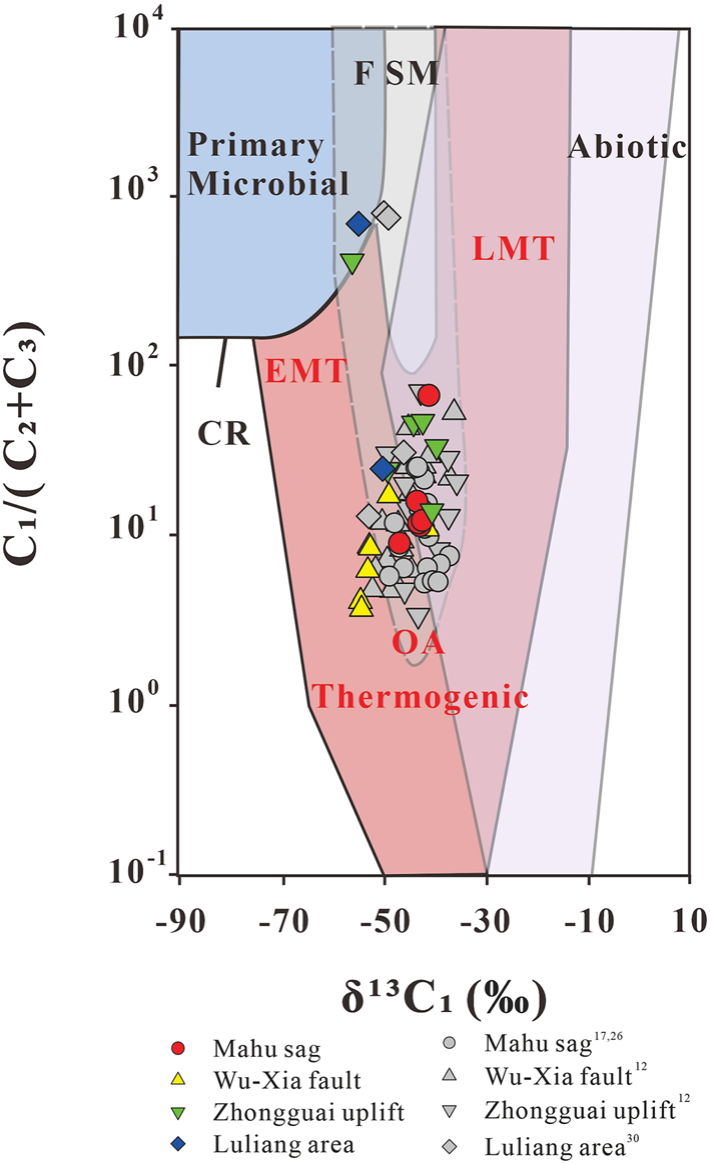
Plot of C_1_/(C_2_ + C_3_) versus δ^13^C_1_ on the genetic diagram of /(from ref.^2^). CR: CO_2_ reduction; F: fermentation; SM: secondary microbial; EMT: early mature thermogenic gas; OA: oil associated thermogenic gas; LMT: late mature thermogenic gas. Data are from this study, refs.^12,17,26,30^.

**Figure 4:**
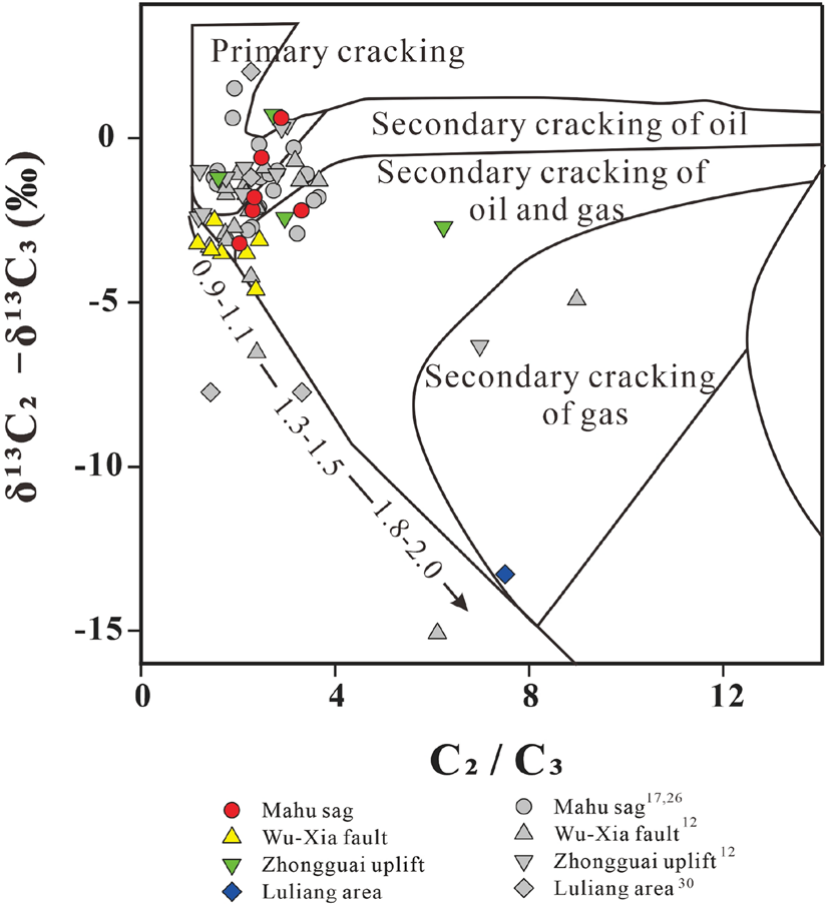
Plot of δ^13^C_2_ -δ^13^C_3_ versus C_2_/C_3_ on (from ref.^34^) with data from this study and refs.^12,17,26,30^.

**Figure 5:**
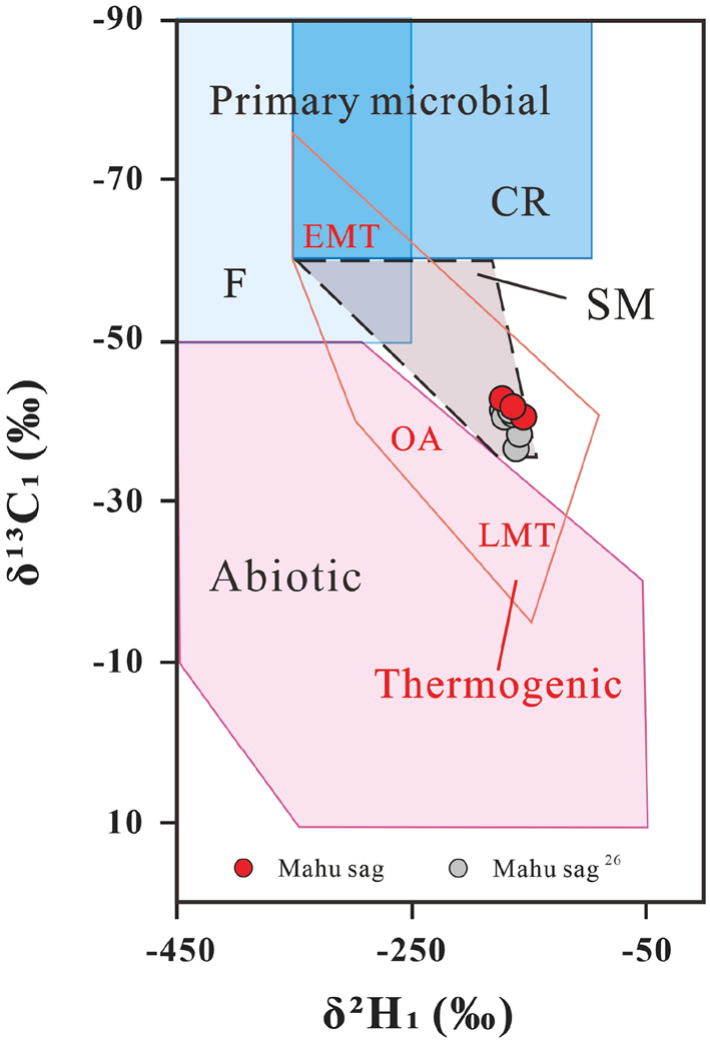
Plot Mahu sag gases on the diagram of δ^13^C_1_ versus δ^2^H_1_ to identify the genetic type (ref.^2^). CR: CO_2_ reduction; F: fermentation; SM: secondary microbial. Some data are from ref.^26^.

**Figure 6:**
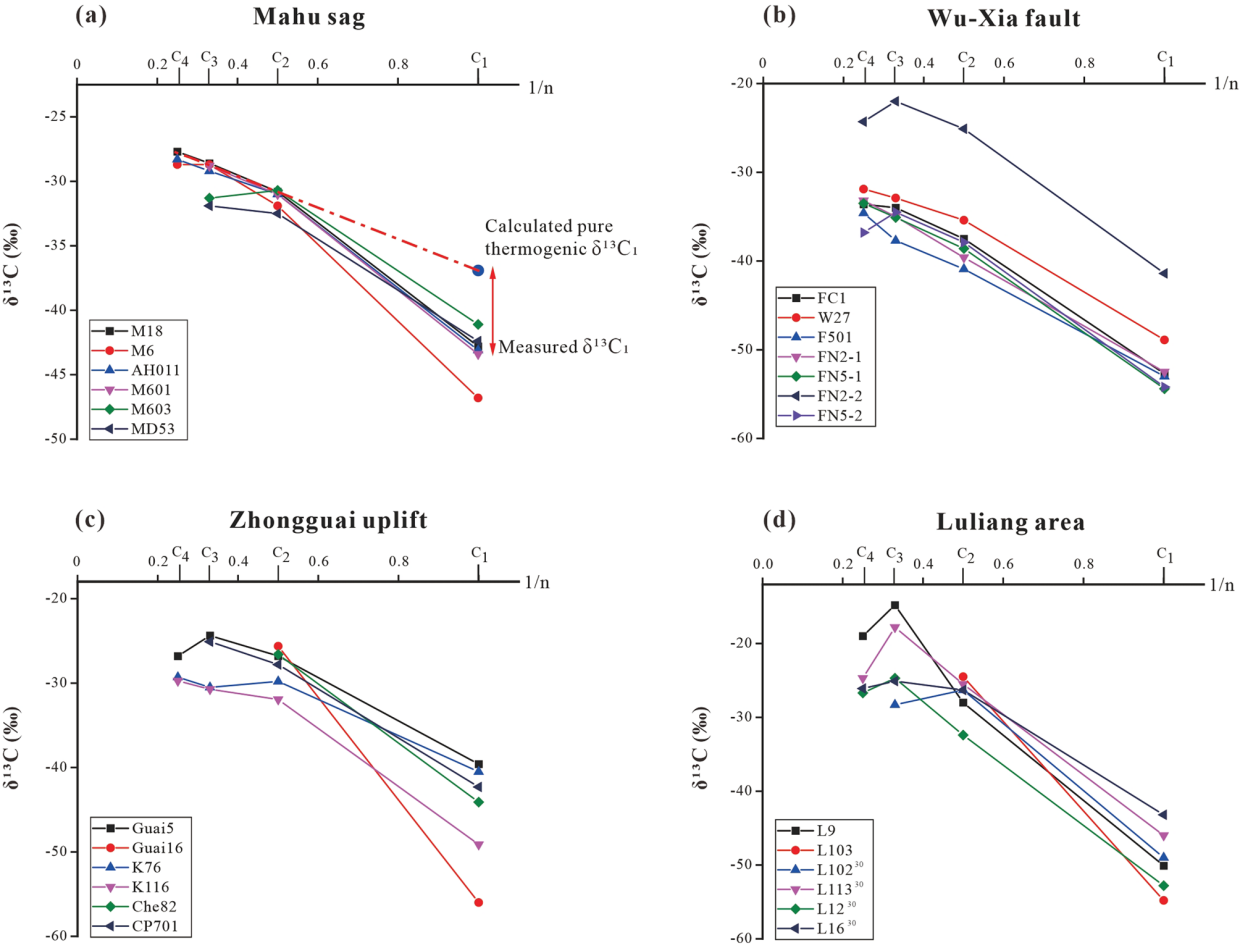
Diagrams of 1/C_n_ versus δ^13^C for gas in Junggar basin. Some data of the Luliang area are from ref.^30^.

The carbon isotope composition of natural gas can be used to indicate the origin, type, and maturity of the gas. δ^13^C_1_ value in combination with the ratio “C_1_/(C_2_ + C_3_)” of gas compositions is widely used to identify the origin of the gases (especially of methane) and the possible processes of gas generation^5,31^. The Junggar Basin gases are plotted mainly within the area of “thermogenic gases” with four samples within the microbial gas area (Fig. 3). The gases from Triassic reservoirs of the Mahu sag have C_1_/(C_2_ + C_3_) ratios from 9.06 to 66.74, and δ^13^C_1_ values from − 46.8‰ to − 41.1‰, most of which are indistinguishable from other “thermogenic gases” with only the highest C_1_/(C_2_ + C_3_) ratio outside the range. A thermogenic origin for the gases is supported by the plot of the C_2_/C_3_ ratio vs the differences in δ^13^C_2_ − δ^13^C_3_ (Fig. 4), which shows ethane and propane of the gases were derived mainly from the primary cracking of kerogen with part from oil cracking at Ro < 1.1%. However, it cannot be ruled out for some of the “thermogenic gases” to have mixed with microbial methane as indicated by methane δ^13^C_1_ and δ^2^H_1_ plot which shows the samples plot on the area representing an overlap between microbial gases and thermogenic gases^32^ (Fig. 5). The “natural gas plot”, a plot of the inverse carbon number (1/*n*) of the C_1_–C_4_ components (the x-axis) against isotope ratio values of each component (the y-axis), showed that primary, unaltered gas, derived from a single source plot along a straight line^33^. Thus, the “natural gas plot” can be distinguished a single-source thermogenic gas from a mixed source. When we plot the data analyzed in this and previous data^30^ from the Junggar Basin on the diagram, some of the gases have ethane, propane, and butane plotting along straight lines in the Mahu sag, Wu-Xia fault and Luliang area (Fig. 6). However, the methane was plotted below the lines showing significantly lighter δ^13^C values. This result suggests that the methane was not co-generated with C_2_–C_4_ fraction and may have mixed with ^13^C-poor methane with δ^13^C from − 54.8‰ to − 41.4‰, which is most likely to be microbial although an origin of early mature thermogenic gas cannot be ruled out^5^.

The C_2_–C_4_ isotope data are extrapolated to the y-axis to predict the δ^13^C of the thermogenic methane endmember. The difference between the predicted δ^13^C of thermogenic methane and measured δ^13^C is due to microbial inputs. According to Chung's gas plot model, we can judge whether microbial or early mature gas mixed with thermogenic gas by calculating the difference between the carbon isotope value of methane in pure thermogenic gas (δ^13^C_1, t_) and that of natural methane measured (δ^13^C_1, m_)^35^. The specific calculation methods are as follows where K is the slope of the C_2_ − C_3_ straight line on the natural gas plot:

1 $${\text{K}} = \left( {\delta^{{{13}}} {\text{C}}_{{3}} - \delta^{{{13}}} {\text{C}}_{{2}} } \right)/\left( {0.{33} - 0.{5}0} \right)$$

2 $$\delta ^{{13}} {\text{C}}_{{1,{\text{ t}}}} = {\text{K}}/2 + \delta ^{{13}} {\text{C}}_{2}$$

3 $$\Delta \delta ^{{13}} {\text{C}}_{{{\text{C1}},{\text{ t}} - {\text{C1}},{\text{m}}}} = \delta ^{{13}} {\text{C}}_{{1,{\text{ t}}}} - \delta ^{{13}} {\text{C}}_{{1,{\text{ m}}}}$$

**Figure 7:**
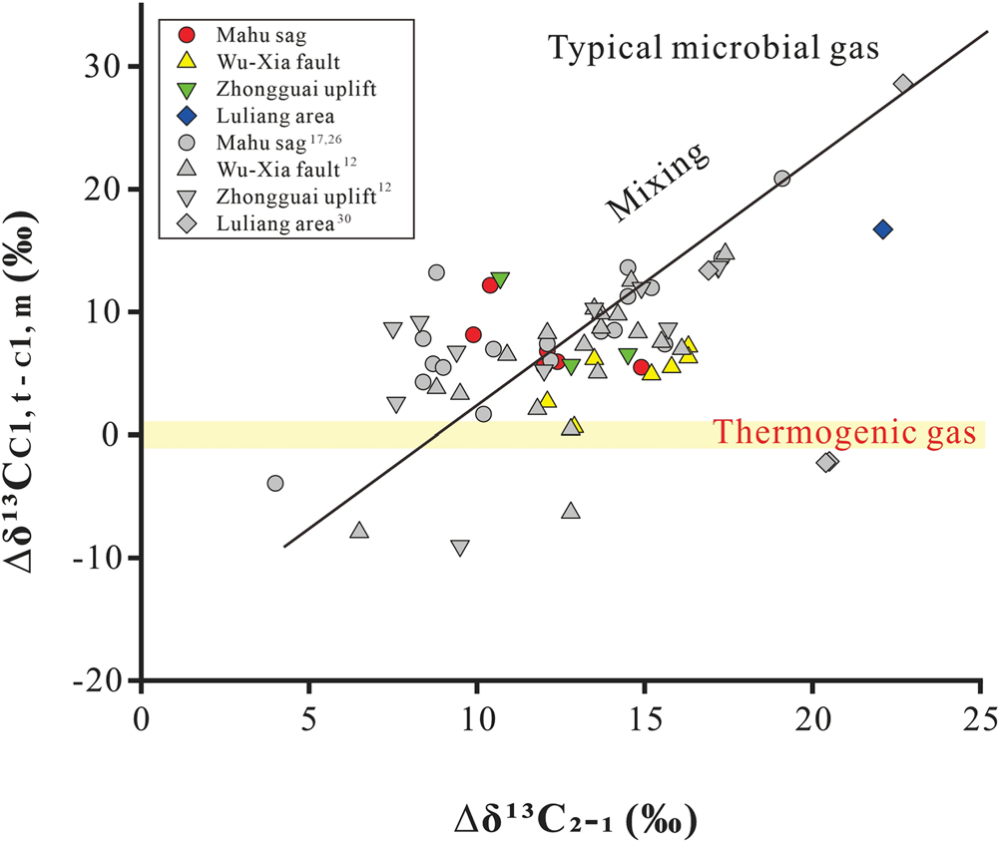
Positive relationship between Δδ^13^C_C1, t‒C1, m_ and Δδ^13^C_2-1_ of natural gas in Junggar Basin with data from this study and refs.^12,17,26,30^.

Besides, we find that the natural gas of Junggar Basin has a positive relationship between Δδ^13^C_C1, t−C1, m_ and Δδ^13^C_2−1_ (Fig. 7 and Supplementary Table S1), indicating that the contribution of more ^13^C-depleted methane to thermogenic gases leads to increase in both the differences. This line of evidence reflects the mixing of gases with ^13^C-depleted methane.

However, recent investigations show that not all the natural gases from a single source are plot along a straight line on Chung’s plot, and some thermogenic gases may show dogleg distribution in C_1_ to C_4_ due to inhomogeneous organic matter components of source rock kerogen^2,35–39^. Thus, other lines of evidence should be present to indicate that at least part of the “thermogenic gases” in Fig. 3 may have mixed with primary or secondary microbial gases.

**Figure 8:**
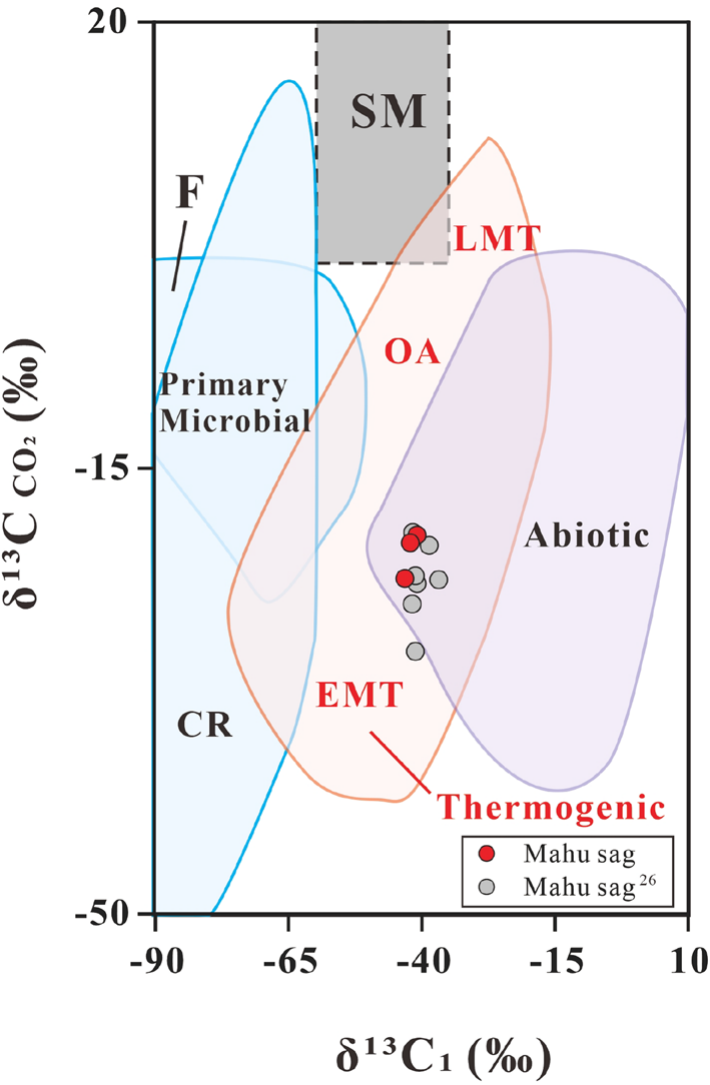
Genetic diagram of δ^13^C _CO2_ versus δ^13^C_1_ (from ref.^2^). CR: CO_2_ reduction; F: fermentation; SM: secondary microbial; EMT: early mature thermogenic gas; OA: oil associated thermogenic gas; LMT: late mature thermogenic gas. Some data are from ref.^26^.

**Figure 9:**
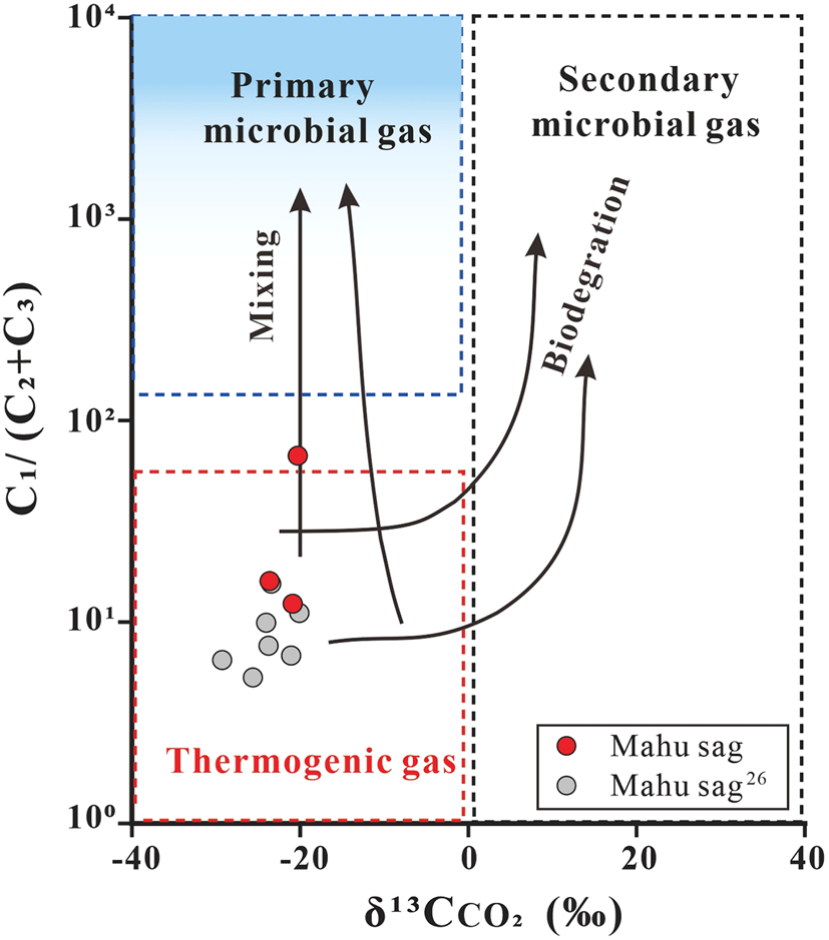
Genetic diagram of C_1_ /(C_2_ + C_3_) versus δ^13^C_CO2_ (from ref.^1^). Some data are from ref.^26^.

Primary microbial gases may have occurred in the Mahu sag. CO_2_/(C_1–5_ + CO_2_) ratio from the Mahu sag T_1_*b* gases shows decrease with δ^13^C_1_^19^. The line of evidence has been considered as the oxidation of methane to CO_2_ in closed systems prior to C_2-5_ charge^19^. This is because methane is the least reactive among saturated hydrocarbons^40^, and thus C_2+_ alkanes are expected to be oxidized preferentially over methane and leave methane intact. On the other hand, as the result of kinetic fractionation, ^12^C-rich methane is preferentially oxidized to ^12^C-rich CO_2_, and when more methane is oxidized, both residual methane and newly generated CO_2_ are expected to be heavier. This newly generated CO_2_ may have precipitated as early calcite cement with δ^13^C values from − 31‰ to − 70‰^18,19^. The oxidation of methane in closed systems are indicated by the positive relationship between MnO content in calcite and the δ^13^C_1_ value of the associated methane^19^. Thus, it can be concluded that the pre-oxidized methane must have δ^13^C_1_ heavier than that of the most ^13^C-depleted Mn-bearing calcite (− 70‰) but lighter than that of the present residual methane (− 48‰). Although thermochemical oxidation of methane have been shown to have fractionation of 16–19‰ based on extrapolation of experimental results at 400–500 °C to 90–135 °C^17^ and a case-study on thermochemical sulfate reduction by methane^41^, microbial oxidation of methane shows a wide fractionation factor between 4 and 30 based upon aerobic culture experiments and model calculation using field data^5,42^. Thus, it is hard to determine the δ^13^C_1_ value of pre-oxidized methane. The present δ^13^C_CO2_ values can be used to differentiate primary from secondary microbial gas, and range from − 29.4‰ to − 20.1‰ in the Mahu sag (Fig. 8). The CO_2_ may be the mixtures among previously existing inorganic CO_2_, methane-derived CO_2_ gas and later charged thermogenetic CO_2_ along with alkanes. Considering that the calcites have more negative δ^13^C values than the associated CO_2_ and a carbon isotope fractionation of < 5‰ during precipitated CaCO_3_ from the original gaseous CO_2_^43^. The methane-derived CO_2_ should have δ^13^C values lighter than the measurement values, which are much lighter than those of secondary microbial gas (> + 2‰). The three aspects of evidence, including, δ^13^C_1_ values of the pre-oxidized methane from − 70‰ to − 48‰, oxidization of only methane in the Mahu sag^17^ and significantly lighter CO_2_ δ^13^C values than the threshold of >  + 2‰ for secondary microbial gas, suggest that the early charged gas must be primary microbial gas. This is supported by the absence of biodegradation of the oils in the association with the gases^17^, one C_1_/(C_2_ + C_3_) ratio higher than the maximum value of the expected thermogenic gases and a trend showing mixing with primary microbial gases (Fig. 9).

By contrast, secondary microbial gases are considered to be generated through the following reactions^44,45^:

$${\text{C}}_{{{16}}} {\text{H}}_{{{34}}} + {\text{16H}}_{{2}} {\text{O}} \to {\text{8CH}}_{{3}} {\text{COO}}^{ - } + {\text{8H}}^{ + } + {\text{17H}}_{{2}}$$

$${\text{CH}}_{{3}} {\text{COO}}^{ - } + {\text{H}}^{ + } + {\text{2H}}_{{2}} {\text{O}} \to {\text{2CO}}_{{2}} + {\text{4H}}_{{2}}$$

$${\text{CO}}_{{2}} + {\text{4H}}_{{2}} \to {\text{CH}}_{{4}} + {\text{2H}}_{{2}} {\text{O}}$$

$${\text{Net reaction}}:{\text{ 4C}}_{{{16}}} {\text{H}}_{{{34}}} + {3}0{\text{H}}_{{2}} {\text{O}} \to {\text{49CH}}_{{4}} + {\text{15 CO}}_{{2}}$$

Secondary microbial gases have been shown in the association with ^13^C enriched CO_2_ and HCO_3_^−^ up to + 32.7‰ and + 26‰, respectively, in San Juan basin^45–48^. Pallasser^47^ and Milkov^1^ found that most of the secondary microbial gases have C_1_/C_1-5_ > 98%, Δδ^13^C_2-1_ > 10‰ and δ^13^C_CO2_ >  + 2‰.

**Figure 10:**
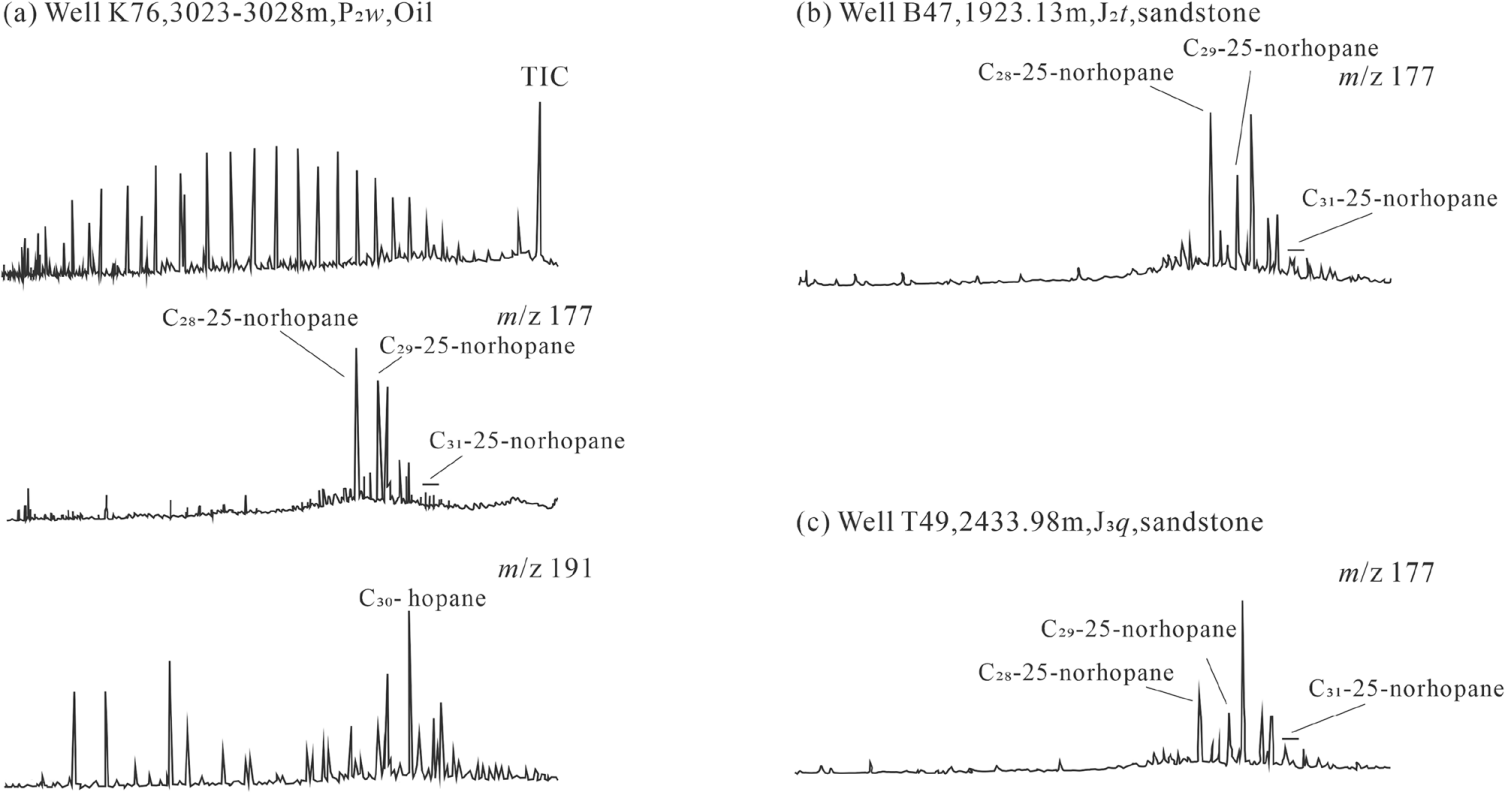
Biomarker chromatograms of, (**a**) a produced oil in the Zhongguai uplift (from ref.^14^), and (**b**) and (**c**) oils extracted from sandstone reservoir in the Beisantai uplift with data from ref.^54^, showing abundant C_25_-norhopanes from biodegraded oils.

Secondary microbial gas may have occurred in the Luliang area, Wu-Xia fault, Beisantai and Zhongguai uplifts. Oil biodegradation is indicated by strong depletion in *n*-alkanes, the occurrence of unresolved complex mixture (UCM), and a series of C_25_-norhopanes from the crude oil in the Permian sandstones from wells K76 in Zhongguai uplift, B47, T49 and DQ-3 in Beisantai uplift and the surrounding (Fig. 10)^20,49–51^. Similar to secondary microbial gases in Australian basins^47^, propane was selectively degraded resulting in a positive shift in its δ^13^C value for some gases from Zhongguai uplift and Luliang and Wu-Xia areas as shown in Chung’s plot with propane plot above the straight lines (Fig. 6b–d). Anoxic degradation of the oils and propane is expected to produce extremely ^13^C-depleted methane in association with ^13^C enriched CO_2_ as the result of carbon isotope fractionation^49,52,53^, thus ferroan calcites from the Permian Wutonggou Formation sandstones from well B-69 in the Beisantai uplift have δ^13^C values of + 22.10‰ to + 22.16‰^20^.

### Primary microbial gas generation and accumulation in the Mahu sag

The source and charge history of the microbial gas are puzzled. Microbial methane is generally accepted to generate from type III kerogen at vitrinite reflectance *R*o < 0.5%. The microbial gas may have charged earlier than thermogenic gas and oil. This proposal is supported by the following two aspects: (1) microbial gas is generated at low temperatures favorable for microorganisms to grow; (2) a calcite with δ^13^C of − 30.6‰ was precipitated at the temperature of 59 °C^19^. That means that the microbial gas was charged at < 59 °C when the reservoirs were buried to < 1300m prior to the late Triassic prior to its oxidation to extremely ^12^C-rich calcite based on burial and thermal history rebuilding^55^. Thus, the primary microbial gases in the Mahu sag are most likely from the P_2_*w* coal-bearing source rock. The source rock was deposited under sulfate-poor freshwater to brackish lacustrine environment with mudstone and shale Sr/Ba ratios from 0.36 to 0.57^56^, and are thus favorable for methanogenesis in the Lower Wuerhe Formation to generate primary microbial gas^57–59^, followed by its up-migration and accumulation in the overlying Lower Triassic reservoirs. The methanogenesis may have occurred prior to the late Triassic when the Lower Wuerhe Formation has organic matter maturity < 0.5% and the underlying Lower Permian Fengcheng Formation, the main source rock for the petroleum in the Mahu sag^12^ experienced temperatures < 70 °C and thus no significant oil and gas has been generated^55^.

### Secondary microbial gas generation in the Zhongguai and Beisantai Uplifts

**Figure 11:**
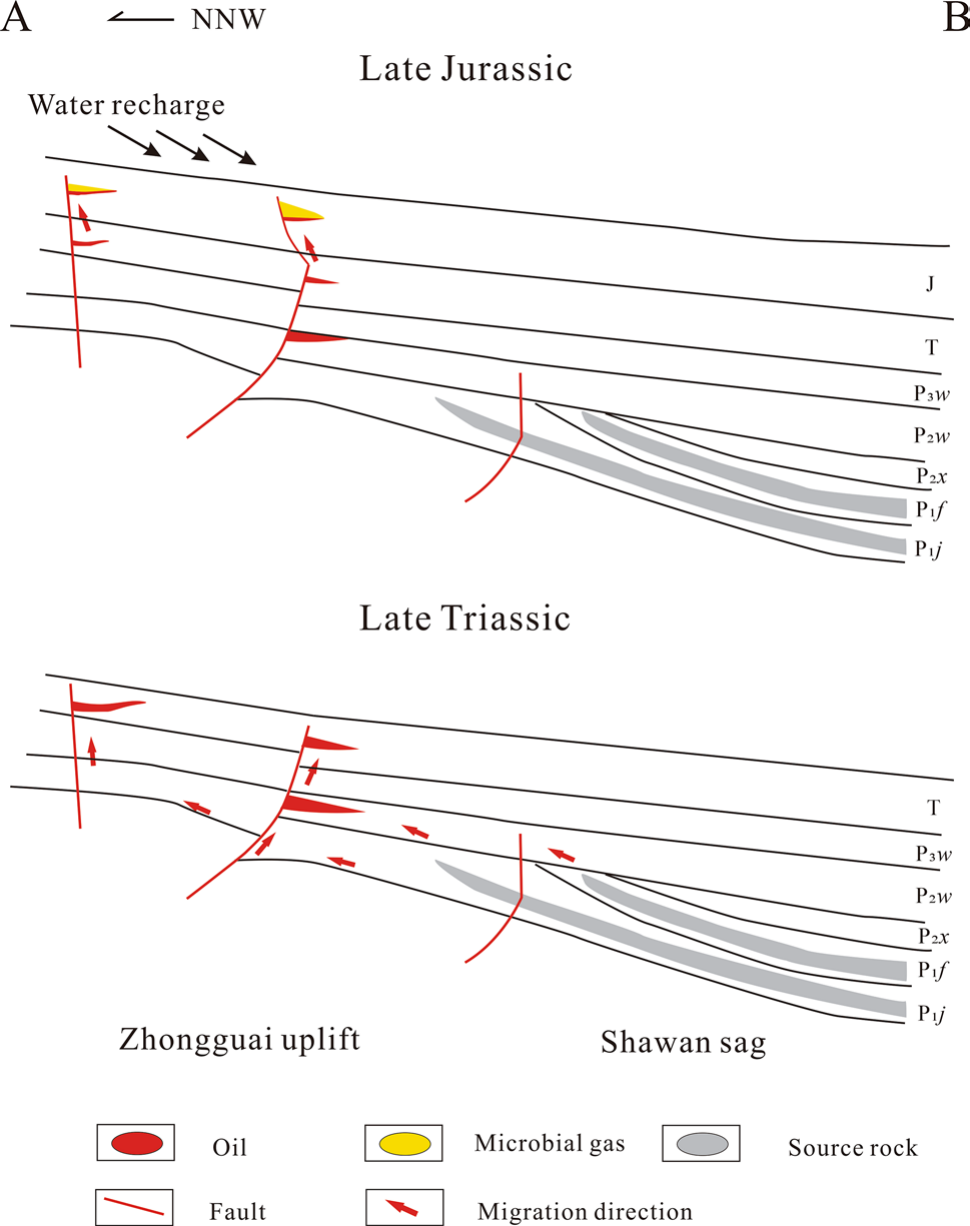
Schematic diagram of the oil and gas accumulation processes in the Zhongguai uplift. The location of the cross-section is shown in Fig. 1.

From the Late Triassic to Early Jurassic, the P_1_*j* and P_1*f*_ source rocks in the Shawan sag reached thermal maturation stage^60^, from which oil and the associated gas generated were then migrated to structural highs and accumulated in the Zhongguai uplift. Oil and the associated gas in Triassic and Middle and Lower Jurassic reservoirs in the Beisantai uplift were generated from the Permian Pingdiquan Formation (P_2_*p*) in the Fukang sag during the Middle and Late Jurassic periods^20^. The oils in both Zhongguai and Beisantai uplifts were sterilized at temperatures higher than around 80–90 °C during deep burial, killing the organisms needed for oil biodegradation to occur^61^. Subsequently, the reservoirs were uplifted significantly to depths with temperatures < 80 °C during the Late Jurassic, followed by the influx of freshwater with bacteria, resulting in biodegradation of oils (Fig. 11) and thus the present oils produced and extracted from sandstones show large unrecognized complex compounds (UCM) and abundant 25-norhopanes (Fig. 10). Biodegradation of oils and propane generated ^13^C-rich CO_2_ and Fe- and Mn-rich calcites and ^12^C-depleted methane-dominated secondary microbial gases in the areas.

## Conclusions

The microbial gas can be concluded to occur from the Carboniferous to the Jurassic in Mahu sag, Zhongguai and Beisantai uplifts. The primary microbial gas in Mahu sag may have generated from the P_2_*w*, type III organic matter with vitrinite reflectance *R*o < 0.5% prior to Late Triassic, and were partially oxidized to calcite with extremely negative carbon isotopic composition and partially mixed with later charged thermogenic gas. Secondary microbial gases from the Zhongguai and Beisantai uplifts were formed from biodegradation of oils and gases from the P_1_*j* and P_1*f*_ source rocks which generated abundant 25-norhopanes and ^13^C-rich CO_2_ precipitating as calcite. The study provides a new case showing how to identify microbial gas in a basin and has implication for microbial gas exploration in the Junggar basin.

### Supplementary Information

Supplementary Table S1.

## Data Availability

The data discussed in this study can be found in the Supplementary Material.
